# Impact of Exercise on Perimenopausal Syndrome: A Systematic Review of Randomized Controlled Trials

**DOI:** 10.7759/cureus.80862

**Published:** 2025-03-19

**Authors:** Ashima Elsa Philip, Hutesh Singh, Shakthi Yogesh Nanjundiah, Petrina Cheryl Samudrala, De Wet Theunissen, Jared Robinson, Indrajit Banerjee

**Affiliations:** 1 Obstetrics and Gynaecology, Sir Seewoosagur Ramgoolam Medical College, Belle Rive, MUS; 2 Surgery, Sir Seewoosagur Ramgoolam Medical College, Belle Rive, MUS; 3 Pharmacology, Sir Seewoosagur Ramgoolam Medical College, Belle Rive, MUS

**Keywords:** climacteric symptoms, exercise, hot flashes, kupperman index, perimenopausal, perimenopause

## Abstract

Exercise has been contemplated as a natural means to alleviate the symptoms of perimenopause. The need for this study arises from the lack of data regarding the impact of exercise on the perimenopausal population. The primary objective of this systematic review of randomized controlled trials was to evaluate the impact of exercise in improving various symptoms of perimenopause, including hot flushes, insomnia, paresthesia, myalgia, arthralgia, palpitations, fatigue, headache, depression, vaginal dryness, and irritability in perimenopausal women. An extensive literature review was conducted according to Preferred Reporting Items for Systematic Reviews and Meta-Analyses (PRISMA) 2020 guidelines, examining the impact of exercise on relieving perimenopausal syndrome. The assessment was based on the readings of the Blatt-Kupperman Index (KI), a scale in which 11 symptoms of perimenopause are rated from 0 (absence of symptom) to 3 (severe). Based on the studies, there was a considerable reduction in the total KI score from baseline by 15.7%. Paresthesia and irritability reduced by 2 points on the scale and showed the greatest improvement. Headache, myalgia, fatigue, hot flushes, and insomnia decreased from an average score of 2 to 1. There was no significant improvement in psychiatric disorders such as depression. Furthermore, its efficacy in treating hot flushes is yet to be established, as discrepancies exist in the results. Exercise has been proven effective in treating mild to moderate perimenopausal symptoms. However, skepticism remains regarding its effectiveness in treating vasomotor symptoms, and further scrutiny is needed to establish it as a viable and effective therapy.

## Introduction and background

Menopause is the permanent cessation of ovarian function, where a woman will no longer experience her monthly menses. It is diagnosed by 12 months of amenorrhea in an age-appropriate female. Perimenopause is the period that precedes menopause and is characterized by a decline in ovarian function. While there is no clear consensus on the prevalence of perimenopausal symptoms, global studies suggest that 22% to 63% of women in Asia, 36% to 50% of women in North America, and 74% of women in Europe experience at least vasomotor symptoms of perimenopause [1], while to varying degrees experiencing symptoms of discomfort such as hot flashes, paresthesias, insomnia, fatigue, arthralgia, myalgia, night sweats, musculoskeletal pain, anxiety, and depression [2]. These symptoms can range from mild to severe and can often interfere with a woman's quality of life. The mainstay treatment for women suffering from these symptoms is most often a form of hormone replacement therapy (HRT) [3]. It helps significantly relieve vasomotor symptoms and vaginal atrophy, and while it is generally considered safe and effective, it does increase the risk of stroke and breast cancer, especially in women above 70 years of age [4], along with a few other risk factors. In comparison, no such side effects have been observed with exercise. An upcoming and possible supplementary treatment, or in selective cases, even an alternative, to conventional HRT could be lifestyle changes, such as physical exercise and dietary modifications. Physiologically, exercise can regulate the endocrine system, including estrogen levels, improve cardiovascular health, reduce cholesterol levels, and decrease waist circumference, all of which counteract the effects of the decline in ovarian function [5-8].

The need for the study arises from the circumstance that there is a lack of data regarding the effect of a non-pharmacological approach such as exercise for perimenopausal syndrome management. The preexisting reviews largely focused on quality-of-life improvement and other varied metrics, such as mood, sleep quality, and fatigue, assessed on various scales, along with a high degree of variability in study design and measurement modalities [9,10]. The primary objective of this systematic review of randomized controlled trials was to evaluate the impact of exercise on improving various symptoms of perimenopause, including hot flushes, insomnia, paresthesia, myalgia, arthralgia, palpitations, fatigue, headache, depression, vaginal dryness, and irritability in perimenopausal women by assessing the improvement and the efficacy of the intervention based on the readings of the Blatt-Kupperman Index (KI), a scale in which 11 symptoms of perimenopause are rated on a scale from 0 (absence of symptom) to 3 (severe). It is vital for such alternative and non-hormonal methods for the amelioration of perimenopausal symptoms to be fully explored, as they may offer a more cost-effective, lower side-effect profile and increased overall health benefits compared to traditional hormone replacement therapy. Furthermore, exercise and dietary modifications need not be used in isolation but can be an adjunct treatment to existing hormonal therapies to further improve the overall response, health, and well-being of a woman suffering from perimenopausal symptoms [11-14].

This paper was awarded 2nd prize in the Best Poster Award at the 4th International Conference on Medical and Health Sciences 2024, held on the 25th Anniversary of Sir Seewoosagur Ramgoolam Medical College in Mauritius on September 16-17, 2024.

## Review

Methodology

This systematic review was conducted under the Preferred Reporting Items for Systematic Reviews and Meta-Analyses (PRISMA) 2020 guidelines.

Literature searches

An in-depth literature search was performed on PubMed, Turning Research into Practice (TRIP), Cochrane Central Register of Controlled Trials (CENTRAL), and Google Scholar databases. The search utilized a combination of keywords, such as Kupperman Index OR perimenopause OR perimenopausal OR menopausal symptoms OR hot flashes OR climacteric symptoms AND exercise. Additionally, Medical Subject Headings (MeSH) terms, including perimenopause, climacteric symptoms, and exercise, were utilized for data extraction (Table 1).

**Table 1:** Databases used for data synthesis

Databases	Boolean Operators	Total Number of Articles
PubMed	Kupperman index OR perimenopause OR perimenopausal OR menopausal symptoms OR hot flashes OR climacteric symptoms AND exercise Filters: 2000-2025	n=3105
Google Scholar	Kupperman index OR perimenopause OR perimenopausal OR menopausal symptoms OR hot flashes OR climacteric symptoms AND exercise	n=1470
Cochrane Central Register of Controlled Trials (CENTRAL)	Kupperman index OR perimenopause OR perimenopausal OR menopausal symptoms OR hot flashes OR climacteric symptoms AND exercise Filters: 01/01/2000 to 14/02/2025	n= 7128
Turning Research Into Practice (TRIP)	Kupperman index OR perimenopause OR perimenopausal OR menopausal symptoms OR hot flashes OR climacteric symptoms AND exercise Filters; 2000 to 2025	n = 370
Total		n=12,073

Inclusion criteria

All randomized controlled clinical trials (RCTs) conducted and published between January 1, 2000, and February 14, 2025, that focused on the effect of exercise on perimenopausal symptoms were thoroughly screened and selected for this systematic review. Only full-text RCTs published in English were included.

Exclusion criteria

Trials investigating the improvement of perimenopausal symptoms that were unavailable or incomplete were excluded. Additionally, non-randomized controlled trials (non-RCTs), cohort studies, case-control studies, cross-sectional studies, abstracts, case reports, editorials, viewpoints, case series, and letters to the editor were excluded.

Data extraction

Data extraction was performed on the selected RCTs. Initially, the abstracts of each study were reviewed, followed by a full-text evaluation of each RCT that met the inclusion criteria. The literature evaluation was carried out by four independent researchers AEP, DWT, PCS, and SYN. The extracted data included study authors, year, design, sample size, study population, control group, pre- and post-intervention KI scores, symptoms with statistically significant improvements, main findings, study limitations, and outcomes.

Risk-of-bias assessment

The ROB 2 tool (Cochrane Collaboration, London, United Kingdom) was used to perform a qualitative assessment of the selected randomized controlled trials. The data were systematically sorted into an Excel sheet (Microsoft Corporation, Redmond, Washington) and entered into the ROBVIS tool to generate a percentage graph (intention-to-treat analysis) as well as a traffic light summary plot. ROB 2 evaluates five domains for each study to determine whether the selected RCTs were at low risk, high risk, or some concern, represented using the colors green, red, and yellow.

Results

Study Characteristics

**Figure 1:**
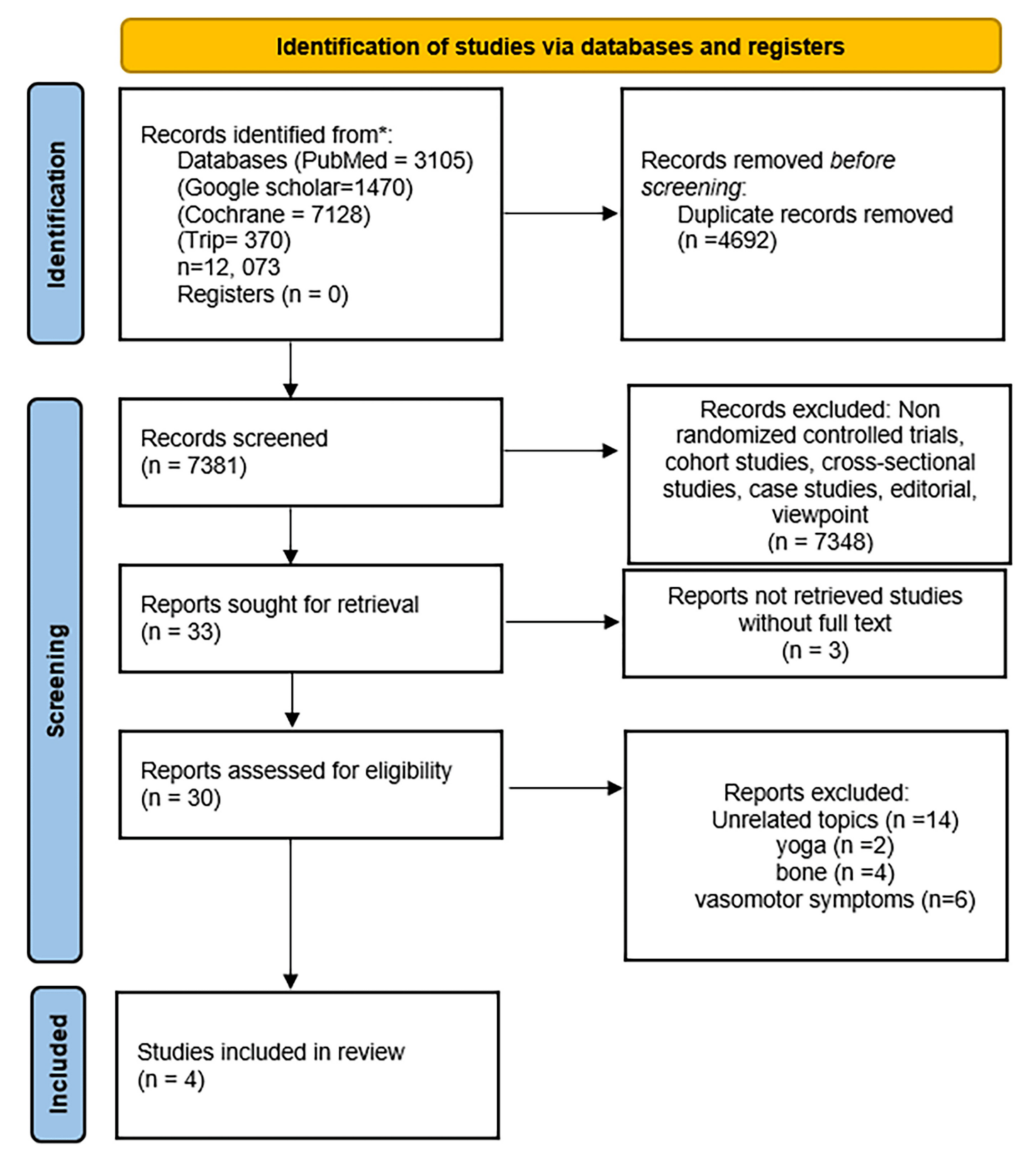
PRISMA 2020 flowchart PRISMA: Preferred Reporting Items for Systematic Reviews and Meta-Analyses.

A thorough literature search was conducted on four databases: PubMed (n = 3,105), Cochrane (n = 7,128), TRIP (n = 370), and Google Scholar (n = 1,470), retrieving a total of n = 12,073 articles. After removing duplicate records (n = 4,692), 7,381 manuscripts remained. Further screening and exclusion of non-randomized controlled trials, cohort studies, cross-sectional studies, viewpoints, abstracts, case studies, and editorials resulted in 33 articles. An additional three studies were excluded due to the unavailability of full-text reports. Subsequently, studies on unrelated topics (n = 14), yoga (n = 2), bone health (n = 4), and vasomotor symptoms (n = 6) were also excluded. Finally, four randomized controlled trials were included in this systematic review for data synthesis (Figure 1).

Risk of Bias Assessments

**Figure 2:**
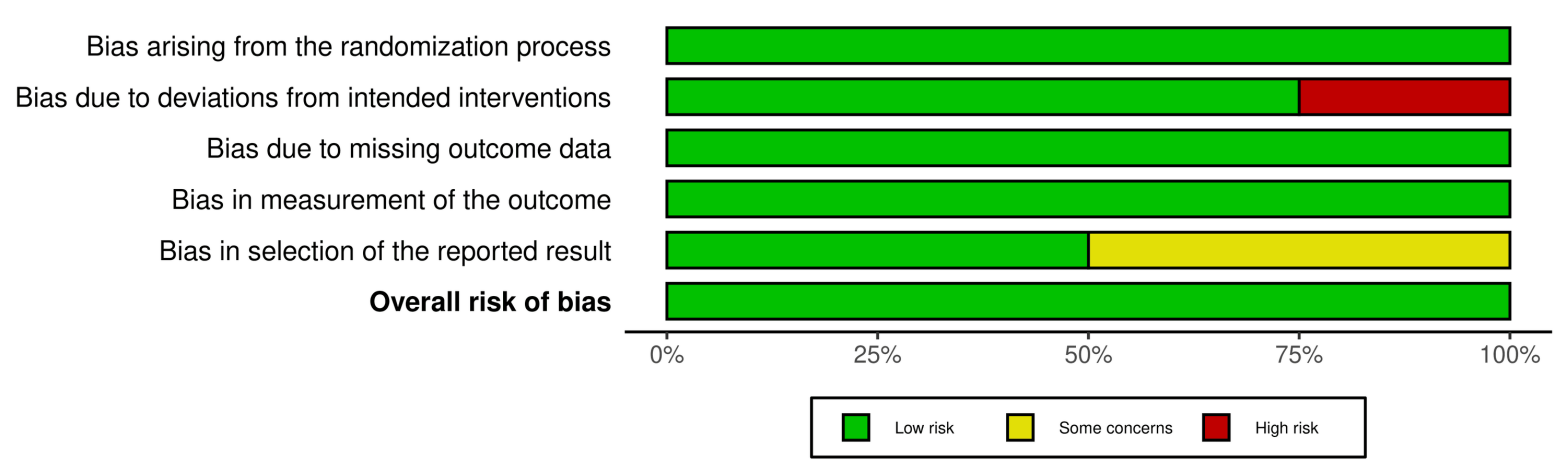
Risk-of-bias assessment: weighted bar plot

**Figure 3:**
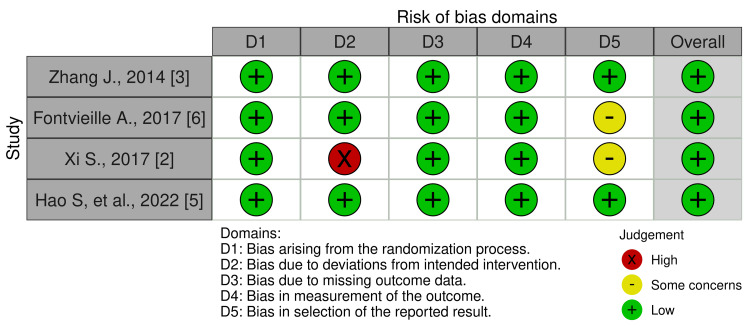
Traffic light plot format of summary of the risk-of-bias assessment

Figure 2 depicts the Risk of Bias analysis weighted bar plot for the selected four randomized controlled trials. Figure 3 shows the traffic light plot for the RCTs.

Synthesis of Findings

Table 2 depicts the authors, year of study, origin of study, duration, sample size, intervention, total KMI pre-intervention, and limitations of the study.

**Table 2:** Summary of study characteristics, interventions, and outcomes KMI: Kupperman menopausal index, BPM: beats per minute.

Authors	Year of Study	Study Design	Origin/Country	Duration	Sample Size	Total KMI Pre-intervention	Intervention	Total KMI Post-intervention (95% CI)	Limitations
Zhang et al. [3]	2014	RCT	Beijing, China	12 weeks	111 subjects	23.7	Walking with strides (aerobic exercise) supervised ≥3 days per week for ≥30 minutes per session	14.5 (13.26–15.74)	Short study duration; Significant dropout rate
Fontvieille et al. [6]	2017	RCT	Quebec, Canada	12 months	31 subjects	15.9	Supervised 3 nonconsecutive sessions per week of aerobic exercise and resistance training for 30 minutes; Phytoestrogen capsules	11.7 (9.13–14.27)	The study lacks a true control group; Participant selection bias; Small sample size
Xi et al. [2]	2017	RCT	Beijing, China	12 weeks	60 subjects	21.8	Any exercise supervised ≥3 days per week for ≥30 minutes per session, target heart intensity: 170 BPM	11.2 (8.40–14.03)	Short study duration; Unblinded study design; Poor generalizability
Hao et al. [5]	2022	RCT	Beijing, China	12 weeks	78 subjects	17.2	2 sessions per week of aerobic exercise and resistance training for 30 minutes depending on the group; Health education	13.5 (10.34–16.66)	Short study duration; Self-reported data

Table 3 depicts the ages of the participants, and clarifies the type of exercise, intensity, and frequency of exercise.

**Table 3:** Authors, age range of participants, type of exercise, intensity of exercise, and frequency of exercise

Authors	Participant Characteristics (Age)	Type of Exercise	Intensity of Exercise	Frequency of Exercise
Zhang et al. [3]	40 to 55 years	Aerobic physical exercise (walking with strides)	Moderate-intensity aerobic exercise	At least three times per week
Fontvieille et al. [6]	50 to 70 years	Resistance training; aerobic exercise	Resistance training: the intensity started at 60% of one repetition maximum (1 RM) and increased to 85% 1 RM during the final 6 months. Aerobic exercise: the intensity was 60-80% heart rate reserve, with training starting at 40-50% and progressing to 70-85%	Three non-consecutive 1-hour sessions per week, consisting of 30 minutes of resistance training and 30 minutes of aerobic exercise
Xi et al. [2]	40 to 55 years	Exercise supervision not specified	Not specified	Exercise supervision twice per week
Hao et al. [5]	45 to 55 years (mean age: 48.65 ± 3.06)	Resistance training (upper limb dumbbell weight series, leg squats/walking squats); aerobic exercise (walking or jogging)	Resistance exercise: moderate intensity, i.e., 30-40 min structured routine with upper and lower body exercises; aerobic exercise; moderate intensity, i.e., walking or jogging 8000-10,000 steps daily	Resistance exercise twice a week; Aerobic exercise; More than 2 days a week

Degree of Improvement in the KI Scores

**Figure 4:**
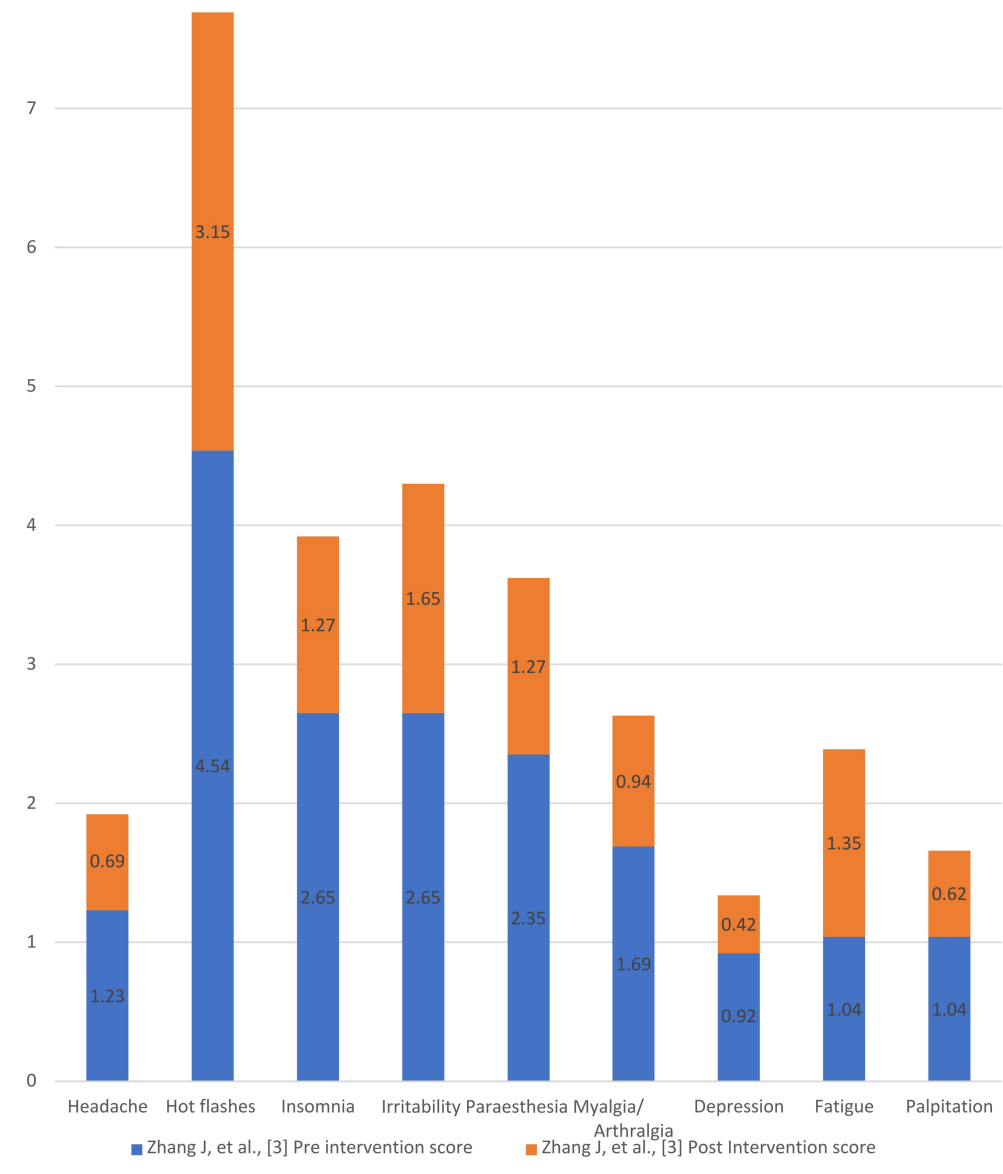
Degree of improvement: pre- and post-intervention Blatt-Kupperman Index (KI) scores on perimenopausal syndrome (Zhang et al.) Data from [3].

**Figure 5:**
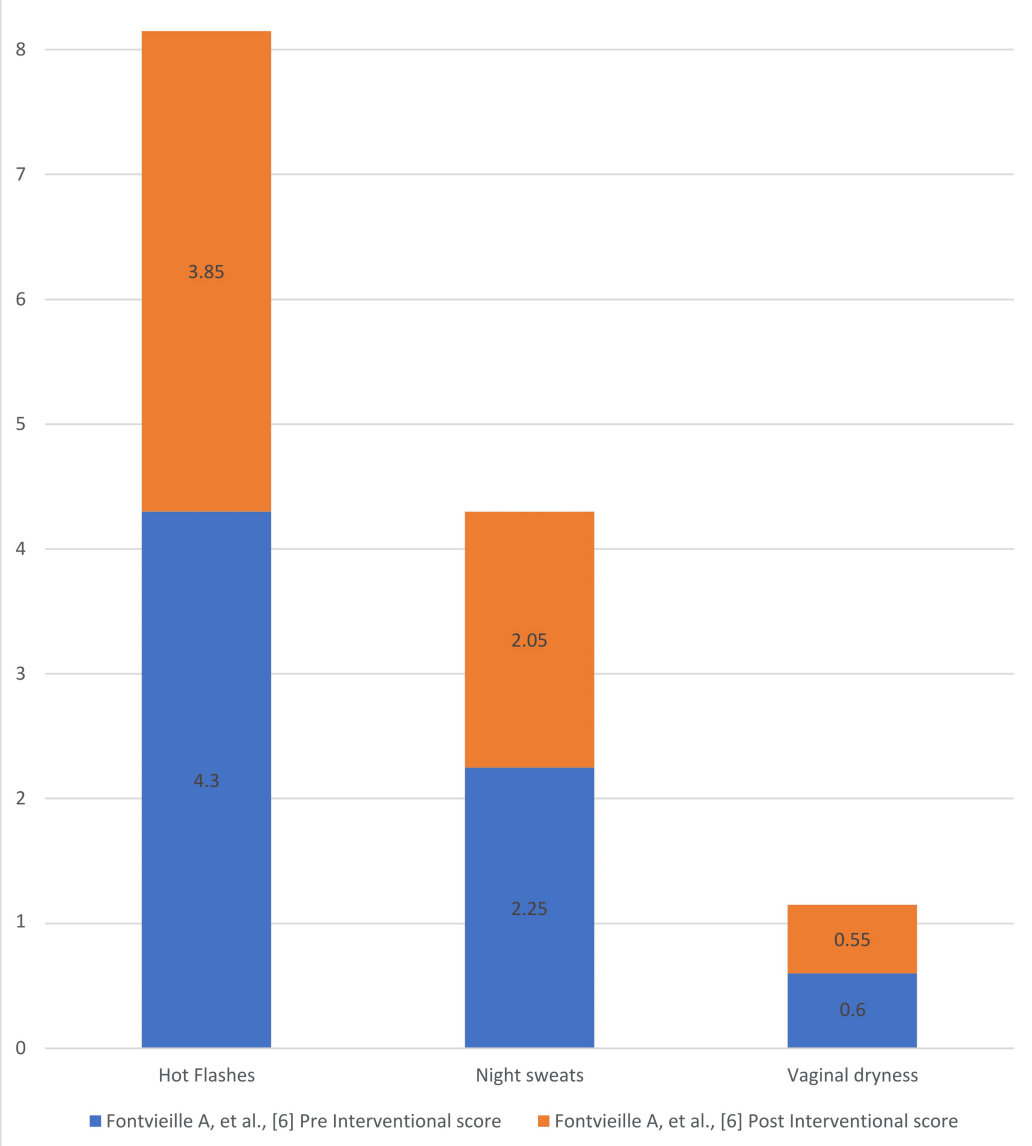
Degree of improvement: pre- and post-intervention Blatt- Kupperman Index (KI) scores on perimenopausal syndrome (Fontvieille et al.) Data from [6].

**Figure 6:**
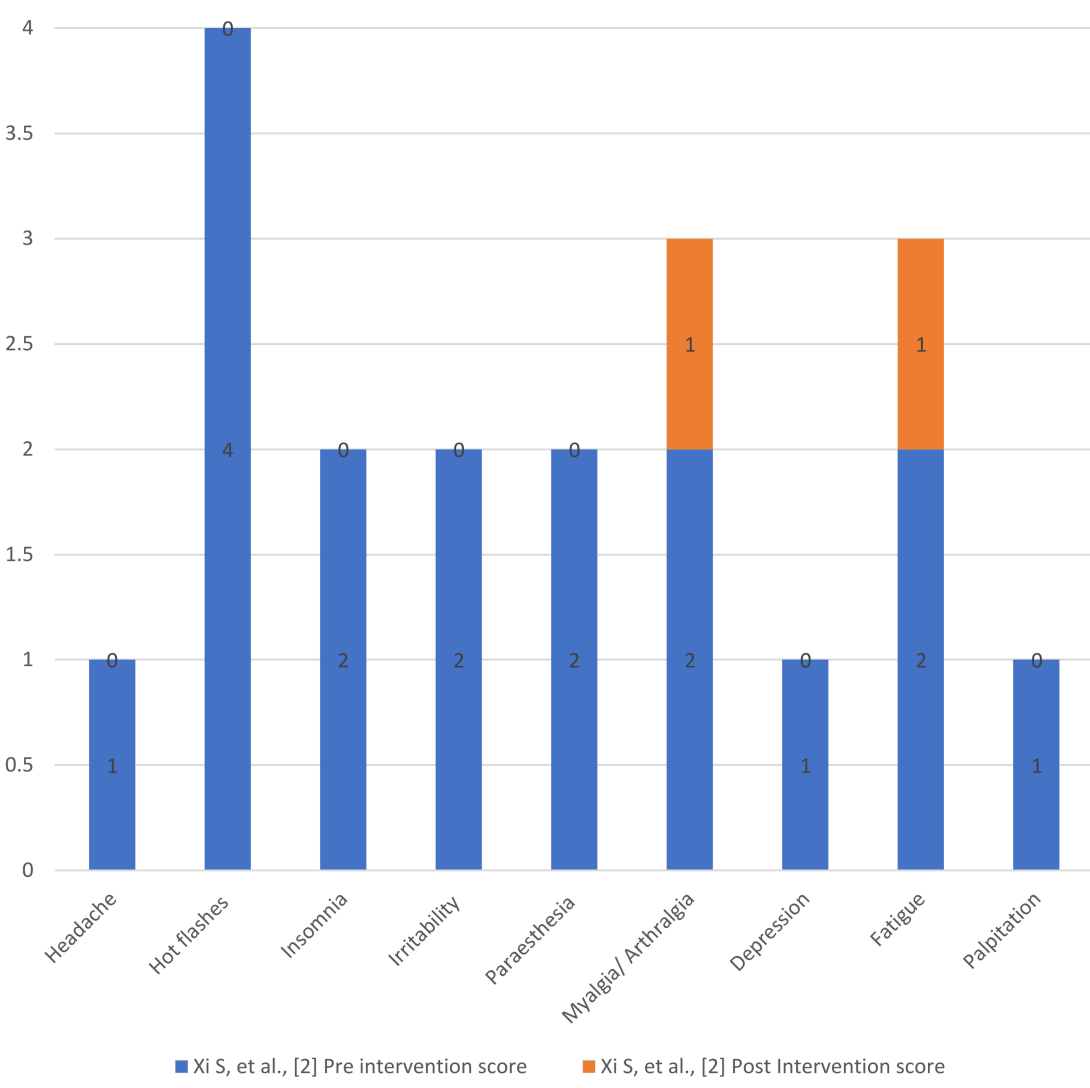
Degree of improvement: pre- and post-intervention Blatt- Kupperman Index (KI) scores on perimenopausal syndrome (Xi et al.) Data from [2].

**Figure 7:**
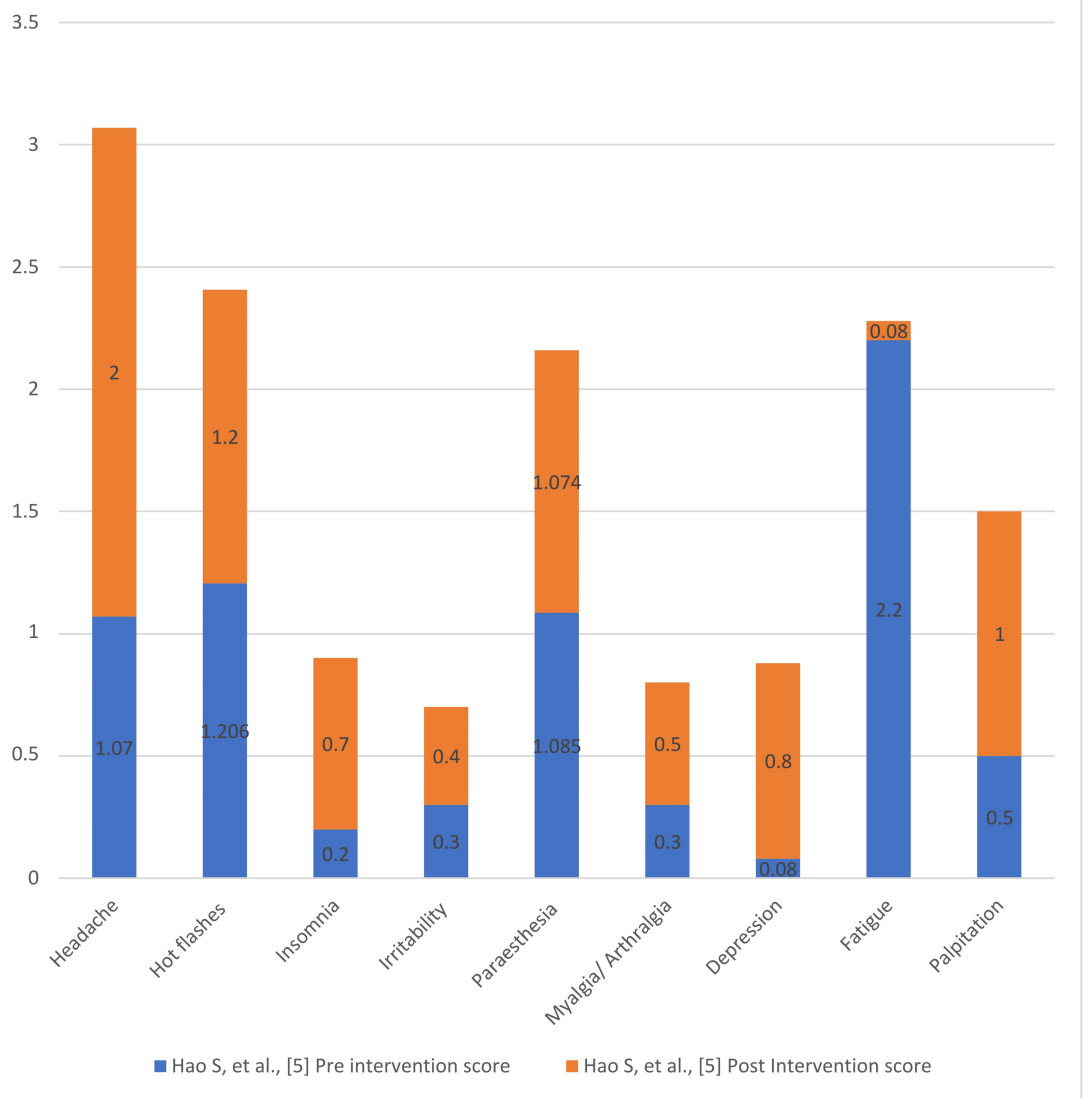
Degree of Improvement: pre- and post-intervention Blatt-Kupperman Index (KI) scores on perimenopausal syndrome (Hao et al.) Data from [5].

Figures 4-7 present the degree of improvement in the KI scores on perimenopausal syndrome after exercise. 

Discussion

Overall, a significant reduction was seen in all the items in the KI, including hot flushes, insomnia, paresthesia, arthralgia, myalgia, palpitations, irritability, night sweats, and vaginal dryness. However, its efficacy in treating hot flushes is arguable.

In a study performed by Xi et al., 60 patients were randomized into either an intervention group (n = 30) or a control group (n = 30). The intervention group’s KI score was taken before and 15 days after the intervention, showing a reduction of 16.37% in total KI scores [2]. There was a reduction in individual scores: symptoms of fatigue, arthralgia, and vasomotor symptoms such as hot flushes and palpitations, as well as mental symptoms such as paresthesia, depression, irritability, and insomnia. In the intervention group, there was also a reduction in the consumption of cereals, meat and poultry, fats, and oils during the 12 weeks, and participants began forming the habit of exercising. Exercise has been frequently linked with reductions in psychological and vasomotor conditions such as depression and hot flushes, respectively [14-16].

In a study conducted by Zhang et al., 157 women were divided into an intervention group (n = 78) and a control group (n = 79). The intervention group performed aerobic exercises three times a week. The study lasted for 12 weeks, during which the reduction in total and individual KI scores was significantly greater in the intervention group than in the control group. The results showed a drastic reduction of 20.97% from baseline, with significant reductions in paresthesia, insomnia, and irritability by an average grade point of 1 [3]. However, the effect of exercise on hot flushes is still debatable and may even be a contributing factor. Exercise was found to be a better alternative to antihypertensive medication due to its less aggravating effects on the body and was used as an adjunct in treating perimenopausal symptoms along with herbal treatments in Chinese women [17-20].

Hao et al. considered a total of 78 perimenopausal female patients, who were randomly divided into three groups: A (centralized education) with 18 cases, B (personalized diet) with 28 cases, and C (personalized diet + resistance training exercise) with 32 cases [5]. The three intervention patterns were performed for 3 months, after which changes in KI scores were observed. From the reduction in total scores, it can be concluded that symptoms of perimenopausal syndrome were alleviated. All three interventions reduced the occurrence of hot flushes, perspiration, excitement, and fatigue. Diet and resistance exercise interventions reduced the occurrence of bone, joint, and muscle pain, headache, palpitations, and sexual disorders. 

The KI score for group A was reduced by 18.96%, highlighting the need for developing effective education strategies regarding perimenopause and how mere awareness empowers patients to take action to reduce symptoms. The insomnia score also decreased significantly after the intervention [15,16]. Group B saw a total reduction of 44.18% in KI scores. Hot flushes and perspiration decreased most significantly, along with five other major symptoms in this group, while the total DASH diet score did not significantly change from pre-intervention levels. Higher consumption of nuts, soy products, low-fat milk, whole grains, poultry, fish and shrimp, white meat, salt, and desserts was observed in this group.

Group C saw a total reduction of 21.60% in KI scores. The symptom scores for hot flushes, perspiration, and sexual disorders were statistically significant. There was an increase in vegetable consumption in group C, while red meat intake was significantly reduced, as reflected in the DASH diet score scale. This suggests that a combination of diet control and exercise has greater potential in reducing symptoms [21-23].

Fontvieille et al. involved 31 women in a 12-month study that included two groups: exercise plus phytoestrogens and exercise plus placebo. The exercise routine consisted of both aerobic and resistance training. It was shown that the total KI score had decreased from baseline by 9.5% in the placebo group and by 0.9% in the phytoestrogen group [6]. The phytoestrogens were of negligible importance, as they did not sustain long-term recovery from perimenopausal symptoms. Additionally, a fraction of the population was unable to break down phytoestrogens, further limiting their effectiveness [24].

Studies point to major improvements in hot flushes, night sweats, and vaginal dryness, showing that exercise plays a crucial role in attenuating vasomotor symptomatology [25,26]. Exercise also had a positive impact on emotional and mental well-being [27]. However, some evident shortcomings included the absence of a sedentary control group to better identify the impact of exercise and the lack of dietary control measures, such as proper standardization of dietary interventions, diet recording, and assessment of equol production, a key factor in phytoestrogen effectiveness.

Another significant drawback was that participants were not stratified based on symptom severity at baseline. This may have resulted in unequal symptom burdens between groups, potentially affecting the results. Improvements such as creating a four-arm study, following controlled diets like DASH, assessing phytoestrogen metabolism, and implementing a longer follow-up duration would help determine whether exercise benefits persist after stopping the intervention.

For future studies, the specific effects of each type of exercise, both aerobic and resistance training, can be analyzed, along with direct comparisons between herbal treatments, pharmaceuticals, and lifestyle modifications to assess their relative efficacy in managing perimenopausal symptoms [28].

## Conclusions

The findings of this systematic review suggest that physical activity over a prolonged period tends to improve the climacteric symptoms of perimenopause. There are notable improvements in symptoms such as hot flushes, paresthesia, irritability, fatigue, arthralgia, and myalgia. Physical exercise is a practical and effective way of managing mild to moderate symptoms of perimenopausal syndrome, particularly for individuals unable to take medication for relief. This finding is relevant as it offers women a non-hormonal method of reducing the debilitating symptoms associated with menopause and perimenopause. Future research can investigate the long-term effects of exercise to assess whether benefits are maintained over time. Additionally, standardization of exercise types and dietary interventions can be studied by maintaining controlled diet plans with proper record keeping of food intake. For studies investigating the effect of phytoestrogens, accounting for individual differences in phytoestrogen metabolism based on equol production might help resolve inconsistencies in findings. Future research can also explore the synergistic effects of exercise, diet, and psychological support on perimenopausal syndrome.
